# In Vitro Assessment of Cytochrome P450 2C19 Potential of Naoxintong

**DOI:** 10.1155/2012/430262

**Published:** 2012-02-16

**Authors:** Hui Chen, Ya Zhang, Xiaoying Wu, Candong Li, Huan Wang

**Affiliations:** ^1^Department of Internal Medicine, Fujian Provincial Cardiovascular Disease Institute, Provincial Clinical College of Fujian Medical University, Fuzhou, Fujian 350001, China; ^2^Clinical Discipline of Chinese and Western Integrative Medicine, Fujian University on Traditional Chinese Medicine, Fuzhou 350108, China

## Abstract

The effects of Buchang Naoxintong Capsules (BNCs) on S-mephenytoin 4′-hydroxylation activities in human liver microsomes in vitro were assessed. Human liver microsome was prepared by different ultracentrifugation. Human liver microsome incubation experiment was carried out to assay BNC on S-mephenytoin 4′-hydroxylation activities. The 4′-hydroxylation of S-mephenytoin, a representative substrate toward CYP2C19, was increased by phenytoin sodium (positive control). After the incubation, the metabolites of the substrates (4′-OH-mephenytoin) were determined by HPLC. Results showed that both phenytoin sodium and BNC showed obvious increase effect on CYP2C19. The enzymatic reaction of BNC was observed with concentrations ranging from 5 *μ*g/mL to 250 *μ*g/mL. Compared to blank, the increase effect of BNC showed significant difference from the beginning of concentration of 150 *μ*g/mL (*P* < 0.001). The conclusion was that BNC showed obvious increase effect on the catalytic activities of drug-metabolising CYP2C19 enzyme.

## 1. Introduction

The efficacy of clopidogrel in combination with acetylsalicylic acid (ASA) therapy has been clearly established in well-designed randomized controlled trials that have showed a reduction in recurrent coronary events following acute myocardial infarction, as compared with ASA monotherapy [1]. However, there is substantial individual variability in response to clopidogrel, with inhibition of ADP-induced platelet aggregation ranging from less than 10% to almost complete inhibition of platelet aggregation with a wide distribution across this range, such that there is no dichotomous separation into “responders” and “nonresponders or resistance” [2]. Such variations in response have repeatedly been associated with adverse cardiovascular outcomes in patients undergoing percutaneous coronary intervention (PCI) [3].

The pharmacology of clopidogrel is key to understanding this phenomenon. Clopidogrel is a prodrug that must be converted to an active metabolite (R-130964). The metabolite inhibits platelet aggregation (the rationale for clopidogrel's use in high-risk cardiovascular disease) by irreversibly binding to the platelet P2Y_12_ adenosine diphosphate receptor. In vivo, 85% of the clopidogrel dose is inactivated by plasma esterases. The remaining 15% is bioactivated in a 2-step pathway that depends on the cytochrome P450 isoenzyme system. The specific isoenzymes involved include cytochrome P450 1A2, 2B6, 2C9, 2C19, and 3A4 [4]. The cytochrome P450 2C19 and 3A4 isoenzymes play the major role.

One mechanism for resistance to clopidogrel involves genetic polymorphisms that alter expression of cytochrome P450 isoenzymes that act on the drug. Of these, CYP2C19*2 is the most common genetic variant reproducibly associated with variability in clopidogrel active metabolite bioavailability, antiplatelet effects, and clinical outcomes [5, 6]. Another mechanism for clopidogrel resistance is competitive inhibition of cytochrome P450 isoenzymes needed for the metabolic activation of clopidogrel. To overcome deficits in clopidogrel responsiveness, someone suggested the addition of CYP inducers to enhance clopidogrel conversion [7]. Our prevenient study found that adjunctive Buchang Naoxintong Capsules (BNC) to clopidogrel can enhance the antiplatelet effect in volunteers with the CYP2C19*2 gene mutation [8]. In the present study, the effect of BNC on the 4′-hydroxylase activity of S-mephenytoin human liver microsome in vitro was assessed.

## 2. Materials and Methods

### 2.1. Drugs

BNC 0.4 g (Compilation of The National Standard of Chinese Traditional Medicine no. WS-10001 (ZD-0001)-2002; Med-drug Permit no. Z20025001) were supplied by the Buchang Pharmaceutical Co. Ltd. The ingredients of BNC include Radix Astragali, Radix Angelicae Sinensis, Radix Paeoniae Rubra, Radix Salviae Miltiorrhizae, Rhizoma Chuanxiong, Semen Persicae, Flos Carthami, Resina Olibani, Myrrha, Caulis Spatholobi, Radix Achyranthis Bidentatae, Ramulus Cinnamomi, Ramulus Mori, Pheretima, Scorpio, and Hirudo.

BNC was flayed, triturated, quantified, and then was dissolved in dimethylsulfoxide (Tianjin Fuchen Chemical Reagents Factory, concentration less than 1/1000).

### 2.2. Human Liver Microsomes

The ten human liver samples were obtained from patients who underwent a partial hepatectomy at the Department of Hepatobiliary Surgery, Fujian Provincial Hospital (Fuzhou, China). Surgery was performed for the removal of liver trauma from the liver. The use of the human liver for the study had been approved by the Institutional Ethics Committee. None of the subjects had a reported history of alcohol or drug abuse. The livers were removed within 2 h, frozen in liquid nitrogen, and stored at −80°C until used for microsomal preparation. Liver microsomes were prepared by different ultracentrifugation as described previously [9]. Liver samples are homogenized and centrifuged at a lower force (9000 r/min) for 15 min. The resulting supernatant is then centrifuged at a higher force (100000 r/min) for 60 min to precipitate the microsomes. The microsomal is resuspended in a final suspension buffer and is then ready for use in incubation studies. Protein and CYP contents were determined using the Bradford Protein Assay Kit (Shanghai Majorbio Bio-Pharm Technology Co. Ltd) and the method of Omura and Sato [10], respectively. The concentration of the protein in liver microsomes was 18 mg/mL, and the total amount of CYP450 enzyme was 589 pmole/mg.

### 2.3. Incubation Conditions

Microsomes (0.5 mg protein) were incubated at 37°C for 60 min with 20 *μ*L S-mephenytoin (250 *μ*mol/L, sigma company, black group) and an NADPH (Sigma-Aldrich Co. Ltd, Shanghai) generating system in the presence or absence of 20 *μ*L BNC (trial group) or 20 *μ*L phenytoin sodium [an inducer of the CYP2C19 enzyme (http://drugs.medsort.com/), sigma company, 15 *μ*g/mL (0.3 *μ*g/mL as the incubation final concentration), positive control group] in a final volume of 1 mL. BNC was dissolved in dimethylsulfoxide (Tianjin Fu Chen Chemical Reagents Factory) and added to the incubation mixture of microsomes. The incubation final concentrations of BNC used were 5 *μ*g/mL, 50 *μ*g/mL, 100 *μ*g/mL, 150 *μ*g/mL, 200 *μ*g/mL, and 250 *μ*g/mL. The same volume of dimethylsulfoxide was added to the black group and positive control group. One sample was divided into five tubes. Adding 3 mL cold dichloromethane (Hao Fly Chemical Co. Ltd, Zhengzhou), the reaction was terminated by cooling on ice. 100 *μ*L phenacetin (2.795 *μ*g/mL, sigma company) was added as an internal standard. The mixture was shaken for 5 min, then centrifuged at 2000 g for 10 min. The upper organic phase was transferred to another tube and evaporated to dryness under nitrogen. The residue was dissolved in 100 *μ*L of eluate and 20 *μ*L was injected into Agilent 1200 high-performance liquid chromatography (HPLC, Agilent Technologies Co. Ltd, USA) system.

### 2.4. Determination of 4′-Hydroxymephenytoin

 4′-Hydroxymephenytoin was purchased from Research Biochemicals International (Natick, MA). Determination of 4′-hydroxymephenytoin was carried out by the HPLC method as reported previously [8]. The mobile phase consisted of methyl alcohol, acetonitrile, and water pH value was adjusted to 8.0 by triethylamine (Hou Wang Chemical Co. Ltd, Nanjing) in the proportion of 17/19/64 and was delivered to a Welchrom C18 column (Shiseido Co. Ltd, Tokyo, Japan; 4.6 mm × 250 mm, 5 *μ*m) at a column temperature of 25°C and a flow rate of 1.0 mL min^−1^. The eluate was monitored at a wavelength of 204 nm. The calibration curve was generated by processing the authentic standard substance through the entire procedures. The coefficient of variation for the intraassay and inter-assay was less than 3.75% and 4.41%, respectively.

### 2.5. Statistical Analysis

 Data were analyzed using SPSS (version 16.0, SPSS Inc., America) and expressed as mean and SD. Analysis of variance (ANOVA) was used as statistical methods to compare group means. A value of *P* < 0.05 was considered to indicate statistical significance.

## 3. Results

### 3.1. Chromatogram

**Figure 1:**
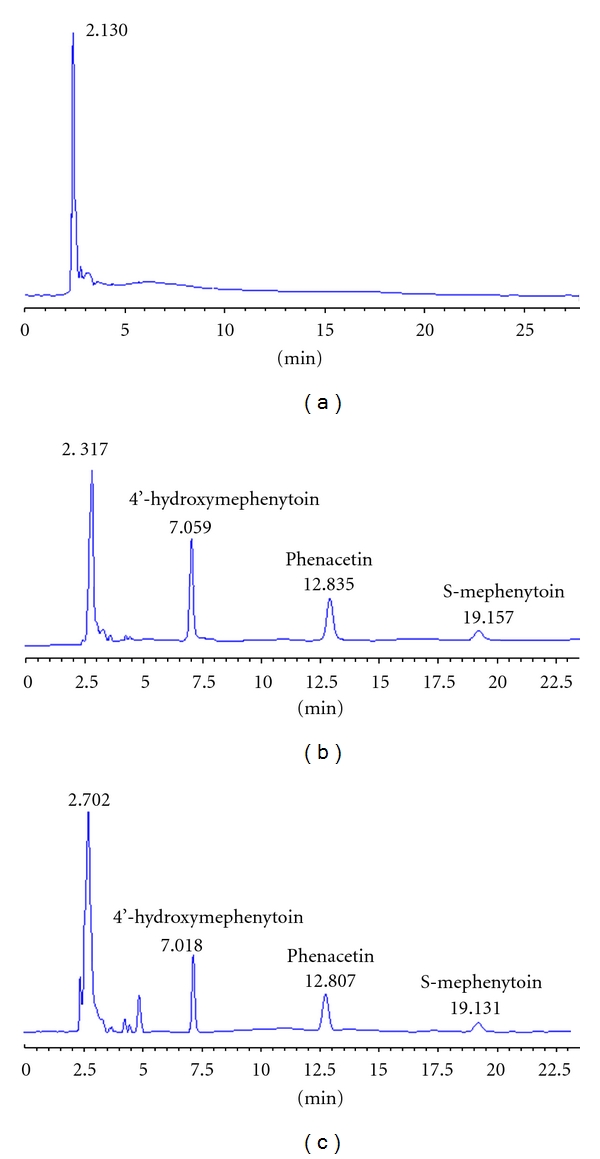
Chromatogram of buffer solutions blank (a), chromatogram of 4′-OH-mephenytoin (7.0 min), phenacetin (12.8 min), and S-mephenytoin (19.1 min (b)), and chromatogram of incubated microsome sample (c), respectively.

 Figures 1(a), 1(b), and 1(c) show chromatogram of buffer solutions blank, chromatogram of 4′-OH-mephenytoin, phenacetin, and S-mephenytoin, and chromatogram of incubated microsomes sample, respectively.

### 3.2. Accuracy

The results from the accuracy studies are give in Table 1.

### 3.3. Recovery Studies (Table 2)

The extraction recovery was given in Table 2. The absolute recovery of 4′-OH-mephenytoin at the three levels of 230, 2300, and 5800 ng/mL ranged from 96.3 to 98.2%.

### 3.4. Linear Range and Detection and Detection Limit (Figure 2)

A linear calibration graph was obtained for 4′-OH-mephenytoin in the range 70.5–5800 ng/mL with a correlation coefficient (*r* ^2^) of 0.9969. The regression equation was written as *Y* = 2678.1*X* + 93.9. This method had a limit of detection of Ca. 39.1 ng/mL.

### 3.5. The Effects of BNC on S-Mephenytoin 4′-Hydroxylation Activities in Human Liver Microsomes

Compared with blank group, the amount of 4′-OH-mephenytoin was significantly increased in positive control group and trial group after preincubated with phenytoin sodium (0.3 *μ*g/mL, *P* < 0.000) and BNC from the beginning of concentration of 150 *μ*g/mL (*P* < 0.001, Table 3) in human liver microsomes. The production rates of 4′-OH-mephenytoin in black group were defined as 100%, these production rates of 150 *μ*g/mL, 200 *μ*g/mL and 250 *μ*g/mL were increased 8.6%, 11.1%, and 12.9%, respectively.

## 4. Discussion

The active metabolite (R-130964) of clopidogrel is a secondary metabolite, and multiple cytochrome P450 enzymes (CYPs), including CYP3A, CYP2C19, CYP2C9, CYP2B6, and CYP1A2, contribute to the two sequential metabolic steps resulting in the formation of R-130964. Of these, CYP2C19 is responsible for approximately 45% of the first step (the formation of 2-oxo-clopidogrel) and approximately 20% of the final step—the generation of the pharmacologically active thiol metabolite. CYP2C19 also is a major metabolizing enzyme of several clinically important drugs such as proton-pump inhibitors (PPIs) like omeprazole and lanzoprazole, antiepileptics-like mephenytoin, diazepam, and selective serotonin reuptake inhibitors like citalopram. PPIs are often given concomitantly with clopidogrel to minimize the chances of gastrointestinal bleeding in patients with acute coronary syndrome especially percutaneous coronary interventions. Recent attention has been placed on a potential interaction observed between clopidogrel and the widely used PPIs. Some evidence suggested that omeprazole interacted with clopidogrel, reducing clopidogrel antiplatelet effects as measured by various laboratory tests [11, 12]. Most data indicated that the interaction involves the competitive inhibition of the CYP2C19 isoenzyme. The interaction appears to be clinically significant, as several retrospective analyses have shown an increase in adverse cardiovascular outcomes when PPIs and clopidogrel are used concomitantly [13–15]. 

Bent evaluated in vitro the dose-dependent induction potential of six commonly used trade herbal products on CYP2C19 and CYP2E1 metabolic activities in cultured human hepatocytes. They found that St John's wort was the most potent CYP-modulating herb, showing a dose-dependent induction/inhibition of CYP2C19, with induction at low dosages and inhibition at higher ones [16]. Previous investigations in man have shown that CYP2C19 activity is susceptible to induction by herbs and natural products; examples include St John's wort, G. biloba and the Chinese herbal mixture Yin Zhi Huang (also called Jaundiclear) [17–19]. Induction of cytochrome P450 isoenzymes, leading to an enhanced platelet inhibitory effect of clopidogrel, has also been described, which suggests a means for overcoming clopidogrel resistance. In a small prospective study, administration of St John's wort enhanced the platelet inhibitory effect of clopidogrel in volunteers known to be unresponsive to the drug and in patients with stable coronary artery disease [20]. Their further studies suggested that St John's wort on the pharmacodynamic response of clopidogrel in hyporesponsive volunteers and patients could increase platelet inhibition by enhancement of CYP3A4 metabolic activity [21]. Our study showed that BNC showed obvious increase effect on the catalytic activities of drug metabolism CYP2C19 enzyme. The increase effect of BNC was from the beginning of concentration of 150 *μ*g/mL. This result might explain why adjunctive BNC to clopidogrel can enhance the antiplatelet effect in volunteers with the CYP2C19*2 gene mutation. It was important clinical meaning. In China, BNC is an approved traditional Chinese medicine (TCM) for stroke [22], which is widely used, and is well tolerated. BNC combined with aspirin could enhance the antiplatelet effect in patients with cardio-cerebrovascular diseases [23], but there is a lack of study of possible drug-herb interactions. Our current study showed that BNC combined dual antiplatelet therapy (DA, clopidogrel plus ASA) enhanced the anti-microembolization (CME) effect of either therapy alone and reduced the risk of the DA therapy-associated bleeding, demonstrating an improved benefit/risk ratio in the rat model of CME by inhibiting platelet aggregation and myocardial apoptosis, balance the pro- and anti-inflammatory cytokines as well as serum ET-1 and eNOS [24, 25]. This result suggested that integrated Chinese and Western medicine might provide a multitarget therapy with potential superior therapeutic efficacy and a better safety profile.

Understanding drug interactions that impair or increase therapeutic efficacy is important, especially with multidrug treatment. Although our findings are provocative, future studies designed to investigate the BNC on CYP2C19 metabolic activities in vivo experimental methods.

## 5. Conclusions

BNC showed obvious increase effect on the catalytic activities of drug metabolism CYP2C19 enzyme. The effect of BNC begins from the beginning of concentration of 150 *μ*g/mL.

## Figures and Tables

**Figure 2:**
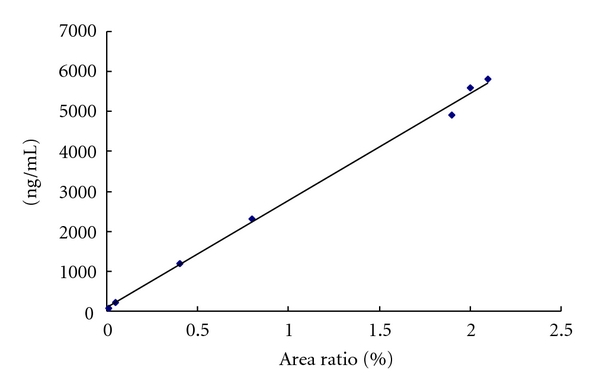
Standard curve of 4′-OH-mephenytoin.

**Table 1:** Precision of the determination of 4′-OH-mephenytoin (mean ± SD, *n* = 5).

Joined concentration (ng/mL)	Intraday (ng/mL)	RSD (%)	Interday (ng/mL)	RSD (%)
230	227.00 ± 8.52	3.75%	227.83 ± 10.05	4.41%
2300	2278.30 ± 58.60	2.57%	2269.30 ± 77.19	3.40%
5800	5849.30 ± 122.44	2.09%	5779.30 ± 141.53	2.45%

**Table 2:** The extraction recovery of 4′-OH-mephenytoin (mean ± SD, *n* = 5).

Joined concentration	Extraction recovery (%)	RSD (%)
(ng/mL)		
230	98.2 ± 4.3	4.4%
2300	96.3 ± 3.9	4.0%
5800	97.7 ± 2.541	2.6%

**Table 3:** Comparison of the amount of 4′-OH-mephenytoin among blank group, positive control group, and trial group.

	Blank group (*n* = 5)	Positive control group (phenytoin sodium, *n* = 5)	Trial group (BNC, *n* = 5)					
Incubation final concentration (*μ*g/mL)		0.3	5	50	100	150	200	250
4′-OH-M (nmol/mg·h)	6.8 ± 0.3	7.7 ± 0.3	6.6 ± 0.4	6.8 ± 0.3	7.1 ± 0.3	7.3 ± 0.4	7.5 ± 0.3	7.6 ± 0.2
		▲▲▲				▲▲,△△,♀	▲▲,△△△,♀♀,†	▲▲▲,△△△,♀♀♀,††

Note: values are presented as mean *± *SD.

▲▲▲, △△△, ♀♀♀: compared with blank, 5 *μ*g/mL, 50 *μ*g/mL, 100 *μ*g/mL, respectively, *P* < 0.000.

▲▲, △△, ♀♀, ††: compared with blank, 5 *μ*g/mL, 50 *μ*g/mL, 100 *μ*g/mL, respectively, *P* < 0.001.

♀, †: compared with 5 *μ*g/mL, 50 *μ*g/mL, respectively, *P* < 0.05.
